# Trichobezoars in Captive-Bred Fat-Tailed Dunnarts and Potential Preventative Protocols

**DOI:** 10.3390/vetsci12070625

**Published:** 2025-06-29

**Authors:** Christine Moschos, Shari Cohen, Emily L. Scicluna, Stephen Frankenberg, Andrew J. Pask, Keshuan Chow

**Affiliations:** 1 School of Veterinary Medicine, Faculty of Science, The University of Melbourne, Melbourne 3010, Australia; 2 Animal Welfare Science Centre, School of Veterinary Medicine, Faculty of Science, University of Melbourne, Melbourne 3010, Australia; 3 TIGRR Laboratory, School of BioSciences, The University of Melbourne, Melbourne 3010, Australia; emily.scicluna@unimelb.edu.au (E.L.S.); srfr@unimelb.edu.au (S.F.); ajpask@unimelb.edu.au (A.J.P.); 4 Office of Research and Integrity, Research Innovation and Commercialisation, University of Melbourne, Melbourne 3010, Australia

**Keywords:** fat-tailed dunnart, *Sminthopsis crassicaudata*, hairball, trichobezoar, enrichment, captive breeding, marsupial

## Abstract

Fat-tailed dunnarts (*Sminthopsis crassicaudata*) are an emerging model species for developmental, reproductive, and conservation biology research. Understanding their husbandry and enrichment needs is integral to ensuring best welfare in captive-bred populations. This was made evident following the sudden deaths of three dunnarts from a population housed at a university research facility between 2018 and 2022. Necropsy results revealed significant trichobezoars causing gastrointestinal obstruction. Following these findings, adjustments were made to the dunnart enclosures with the addition of autoclaved tree bark to capture loose fur as well as the inclusion of paraffin oil in the diet for the purpose of gastrointestinal lubrication. Since these husbandry interventions, no further deaths attributed to trichobezoars have been reported. Here, we present these data and outline new best practice methods for captive dunnart husbandry.

## Simple Summary

Trichobezoars have been reported in numerous species including, but not limited to, humans, cows, cats, dogs, rabbits, ferrets, guinea pigs, and marsupials. In non-human animals, they are caused by an accumulation of hair in the gastrointestinal tract that has been ingested typically through routine grooming. This work describes the presence of trichobezoars occurring in fat-tailed dunnarts and outlines successful preventative methods. The addition of dietary paraffin oil alongside the inclusion of autoclaved tree bark in the dunnart enclosures were preventative protocols implemented to avoid trichobezoar-related deaths in a captive-bred population housed at a research facility.

## 1. Introduction

The fat-tailed dunnart (*Sminthopsis crassicaudata*) is a carnivorous marsupial endemic to Australia and classified as a threatened species in Victoria [1,2,3,4]. It is often utilised by research institutions as a model species to study marsupial reproduction and developmental biology [2]. However, published literature regarding the management and husbandry practices of this species are limited compared to other captive-bred or research animals [5,6,7,8,9,10,11]. There is also a lack of published, grey, or anecdotal literature on the management and incidence of trichobezoars in dunnarts. Methods to avoid recurrence in the colony was sought after the sudden deaths of several individuals over a period of three and a half years. Necropsy of these individuals revealed foreign material in their gastrointestinal tracts resembling trichobezoars.

Trichobezoars, colloquially known as ‘hairballs’, have been reported in numerous species including human and non-human primates [12], cattle [13], domestic cats [14,15], lynx [16], lions [17], tigers [18], rabbits [19,20], ferrets [21], guinea pigs [22], and marsupials [23,24,25]. They occur as the result of excessive fur or hair ingestion that accumulates in the gastrointestinal tract (GIT). Under normal circumstances, the hair is passed through the GIT and expelled from the body in faeces [14]. However, if the quantity of fur ingested is too significant or there are abnormalities in gastrointestinal motility, the fur may accumulate into a solid mass and cause a blockage [15,17,18,20]. Clinical signs of trichobezoars are often non-specific and may have a late onset, which may lead to a delayed diagnosis [17]. Across all species, trichobezoars are associated with decreased appetite, lethargy, weight loss, abdominal pain, and recumbency [12,13,14,18,20]. In some species, trichobezoars may be an incidental finding, while in others, the condition can become fatal [20,21].

The cause of trichobezoars is not well understood but is thought to be multifactorial. The literature suggests that diet can play a significant role [18,20,22,25,26]. Feline studies indicate that fibre can be an important dietary component to reduce the risk of hairball formation [14,27,28]. The presence of trichobezoars in captive Dunnart colonies is occasionally reported in the literature but may be underreported as not all colony animals are necropsied. Therefore, frequency and incidence of trichobezoar occurrence remains unknown. Several studies suggest that the addition of an intestinal lubricant to the diet may also aid in reducing the occurrence of trichobezoars by assisting in movement of hair through the GIT [18,21,22,29]. In other species, brushing the coats of predisposed animals can be beneficial to dislodge excess fur from the body and in turn decrease the amount that is ingested [14]. This is particularly important during spring when seasonal shedding is at its highest [13,20,21,22,28]. Animals that are housed or managed inappropriately (e.g., stocking density, enrichment) can be prone to overgrooming behaviours predisposing them to trichobezoar formation [12,17,29]. Overgrooming is also more likely in animals with a high parasite burden or pruritic skin disease [13,14,17,21,22].

The aim of this study was to highlight the health issues of trichobezoar development in captive fat-tailed dunnarts and to explore management protocols to minimise their formation. Management methods utilised to reduce the frequency of trichobezoar formation were the addition of an intestinal lubricant alongside environmental modifications to enclosures to assist the removal of moulting hair.

## 2. Materials and Methods

### 2.1. Animals

A population of captive laboratory fat-tailed dunnarts were housed at a university research facility. The population consisted of both male and female dunnarts and the population size fluctuated between 63 and 146 individuals over a four-year period from 2018 to 2022. Captive management of animals included in this opportunistic research were approved by The University of Melbourne Animal Ethics Committee (AEC: 1814630) and managed with the appropriate Wildlife Permit (number 10009935) from the Department of Environment, Land, Water and Planning.

### 2.2. Enclosures

The animals were housed in modified rat cages measuring 550 L × 360 W × 200 H (mm) with wire lids. Breeding females were moved to metal glass-fronted cages measuring 420 L × 310 W × 230 H (mm) with side-attached nest boxes (130 × 130 × 130) during pregnancy and weaning. Both enclosures had a soft absorbable paper sheet placed into the bottom of each cage, with an egg carton filled with shredded paper, and cardboard tubes for additional hiding spaces available. The underground indoor rooms were kept between 18 °C and 22 °C and maintained on a 12:12 (light:dark) cycle with standard light bulbs.

### 2.3. Diet

The animals were offered water ad lib. A diet consisting of Wombaroo (Glen Osmond, South Australia, Australia) ‘Small Carnivore Mix’, Advanced kitten dry cat biscuits (Mulgrave, Victoria, Australia) and Royal Canin (Melbourne, Victoria, Australia) or Recovery feline/canine, and Wombaroo ‘The Good Oil’ (Glen Osmond, South Australia, Australia) was fed daily. Each animal received 5–10 g/day, increasing to 30 g/day for pregnant females or those with young. All animals received two portions of 5× meal worms and one portion of 3× crickets per animal weekly as well as a daily ration of Pentavite multi-vitamin for children. The full recipe can be found in Appendix A.

### 2.4. Records

Records dating from 16 April 2018 to 24 March 2022 were stored in electronic spreadsheets. These records included information on the number of male and females in the colony, the number of cages they were kept in, and the number of recorded deaths or culls on any given day. Additionally, information regarding the reason for culling and pathology described in the necropsy was documented. Records were manually sorted to extrapolate a data set of all animals that were culled or found dead over the four-year period.

### 2.5. Necropsies

Necropsies were performed in one of two ways:

1. In-house necropsies were carried out by experienced laboratory technicians and included an assessment of body condition, nutritional and hydration state, pregnancy status, and a systemic evaluation of major body systems.
2. Cadavers were transferred to Cerberus Science, an ISO accredited specialist animal laboratory pathology lab, where qualified veterinary pathologist carried out full necropsies.

### 2.6. Husbandry Interventions

**Figure 1:**
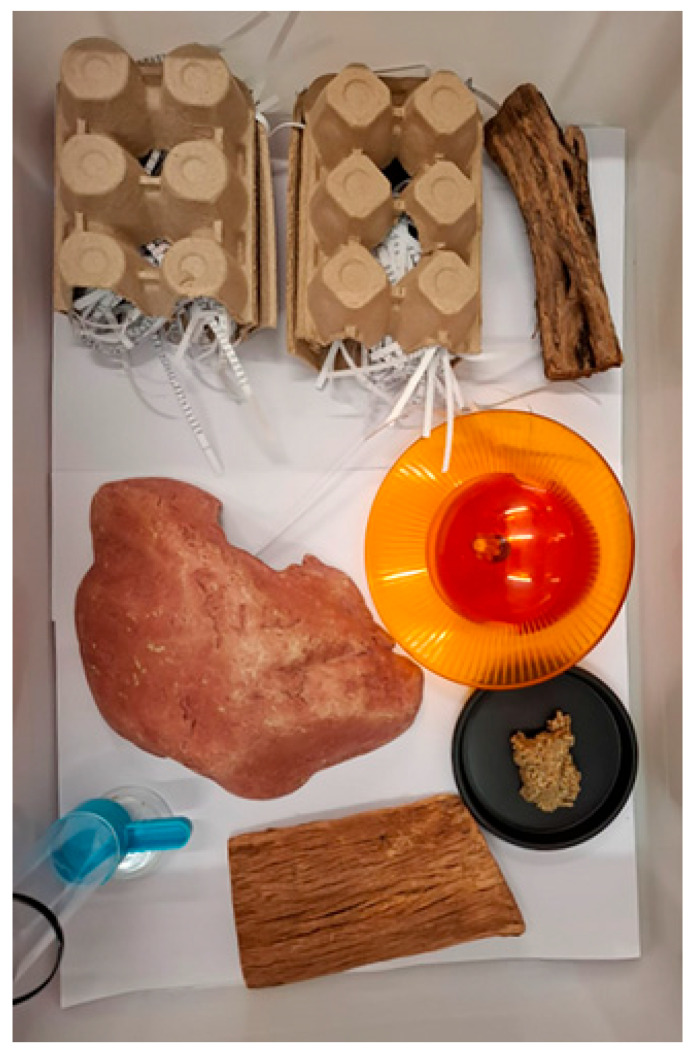
Dunnart enclosure with added enrichment following the husbandry interventions of extra bark and a running wheel. Taken from Scicluna 2025 with permission.

Rough vegetation in the form of autoclaved bark tunnels and logs were added to the enclosures in mid-September 2021 (Figure 1). Approximately one month later during October 2021, the diet was amended to include 50–75 μL of Vetsense Paraffin oil daily per dunnart.

## 3. Results

During the four-year study period, 3 out of 22 deaths were recorded with pathology resembling a trichobezoar (Table 1). However, there were some additional deaths (within the natural attrition rate) where animals were not necropsied. A diagnosis was not determined for cause of death for these latter animals, some of which may have been caused by trichobezoars. The three confirmed case studies with relevant information are outlined below in Table 1.

### 3.1. Animal A (My291)

**Figure 2:**
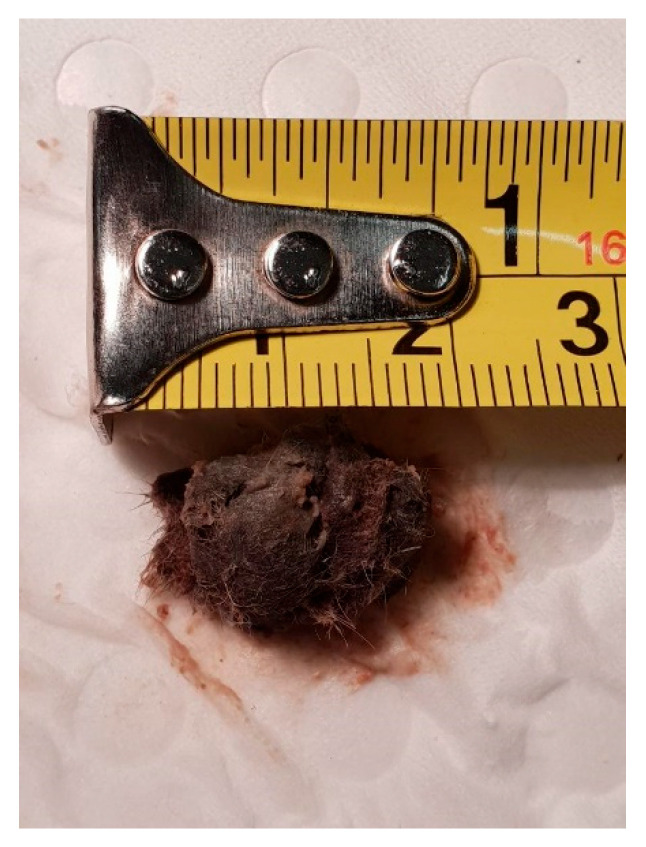
A 22 cm trichobezoar removed from the small intestine of Animal A during necropsy.

**Figure 3:**
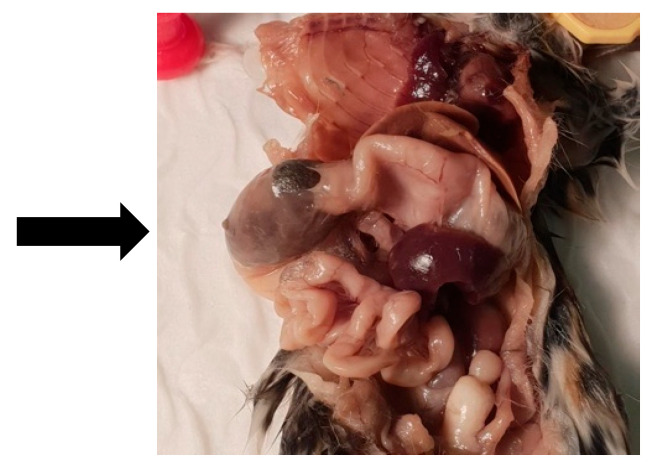
Black arrow pointing to the large mass visible in the small intestine of Animal A with perforation.

A male dunnart, one year and four months old, was found suddenly dead without any known issues on 7 November 2020 (Australian spring). Upon gross dissection, the dunnart’s stomach appeared swollen. Necropsy revealed a 2 cm furball lodged tightly in the small intestine (Figure 2). This appears to have obstructed the whole digestive tract (Figure 3). No food or faecal waste was present in the large intestine or colon. At time of death, there were 85 dunnarts in the population housed across 31 cages.

### 3.2. Animal B (868)

**Figure 4:**
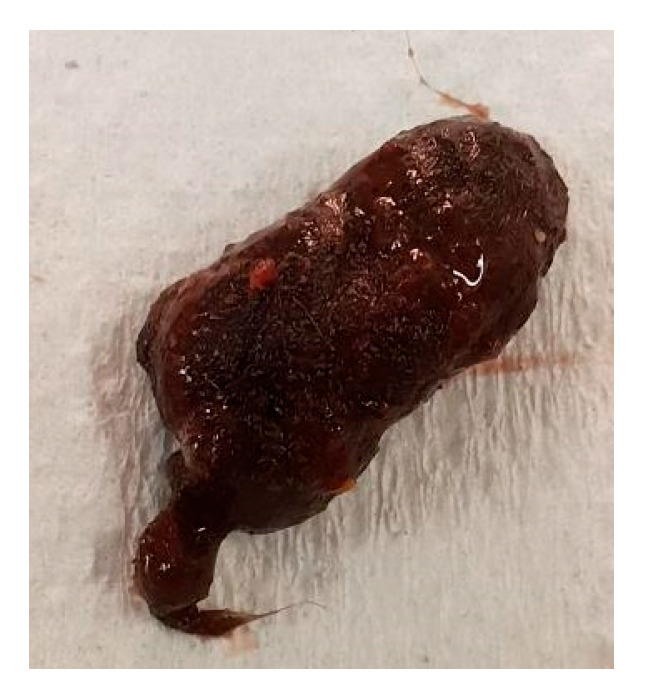
Trichobezoar removed from the gastrointestinal tract of Animal B during necropsy.

A female dunnart, eight months old, was found suddenly dead without any known issues on 8 August 2021 (Australia winter). Necropsy revealed a furball (estimated < 2 cm) considerably distending the gastrointestinal tract (Figure 4). At the time of death, there were 117 dunnarts in the population housed across 38 cages.

### 3.3. Animal C (768)

**Figure 5:**
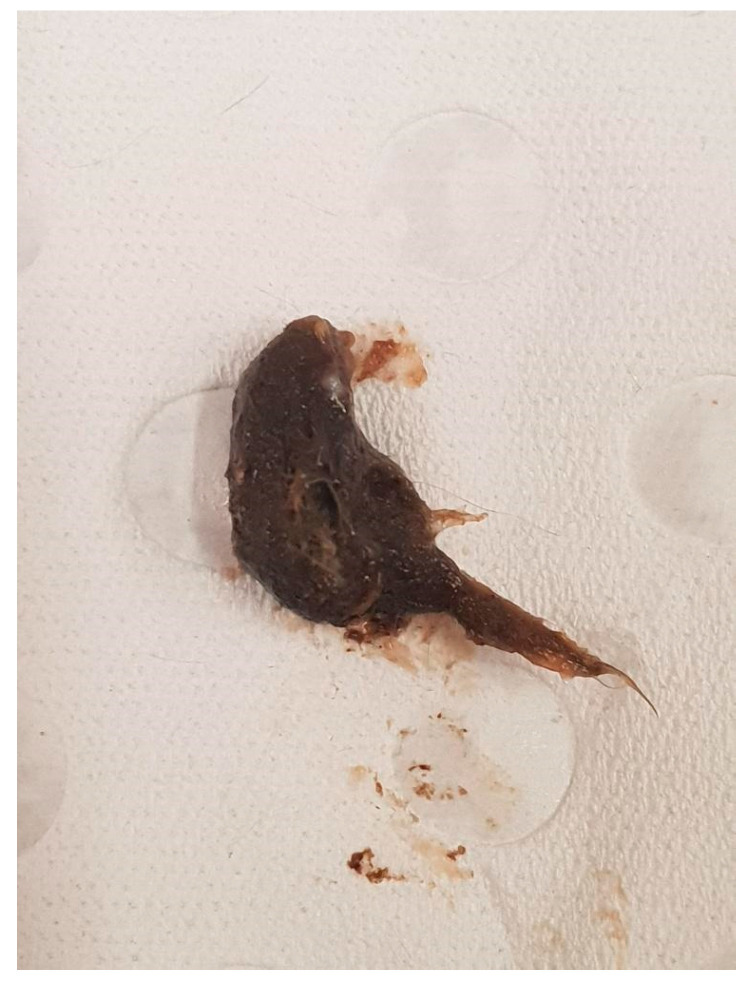
Trichobezoar removed from the gastrointestinal tract of Animal C during necropsy.

**Figure 6:**
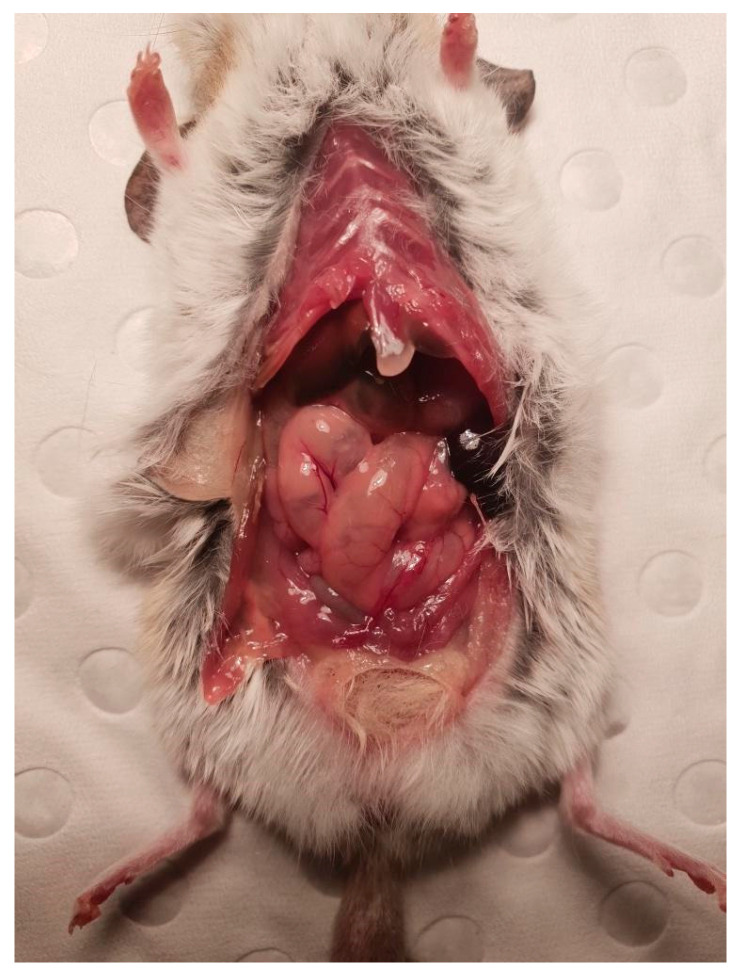
Gross appearance of abdomen of Animal C prior to necropsy with distended intestines.

A female dunnart, one year and seven months old, was found suddenly dead without any known issues on 4 October 2021 (Australian spring). Necropsy revealed a large trichobezoar taking up the entire space inside the stomach causing a blockage to the small intestine tract (Figure 5). There was no food or waste present in the lower gastrointestinal tract below the blockage (Figure 6). At the time of death, there were 106 dunnarts in the population. The female was housed with one other female and one breeding male. The female found dead was not pregnant, nor did she have any young in pouch or at foot at the time of her death.

### 3.4. Post-Intervention Trichobezoar Deaths

Following the preventative management changes in September/October 2021 that have continued as part of routine preventative colony management, no further deaths have been recorded or known to be attributed to trichobezoars since October 2021 within the colony.

## 4. Discussion

There have been published and anecdotal reports of trichobezoars in fat-tailed dunnarts [2]; however, none have attempted preventative protocols. To the authors’ knowledge, this is the first published protocol for the prevention of trichobezoar occurrence in fat-tailed dunnarts. The results of this study signify the importance of appropriate diet and enrichment to prevent future recurrence of cases. This information may be beneficial to this species and other small marsupial populations housed in captivity. This study has identified that the successful ongoing application of an ingestible gastrointestinal lubricant in the feed alongside environmental modifications can possibly reduce the number of deaths or disease caused by trichobezoars.

### 4.1. Diet Considerations and the Use of Gastric Lubricants

Although there is limited information on the underlying cause of trichobezoars, one factor hypothesised is the arrival secondary to gastrointestinal dysfunction [13,15,20,30]. This has been documented in various species and may be a result of inappropriate diets or dehydration [30,31]. The gastrointestinal tract of dunnarts consists of a unilocular stomach and simple intestines similar to that of felines [2,32]. For this reason, the general principles of trichobezoar management were applied to the dunnart colony with an approach similar to what has been successful for domestic cats.

Fixed feeding regimes offered in captivity are suggested to also contribute to the occurrence of trichobezoars [17]. Dunnarts are a carnivorous species and naturally eat a diverse diet consisting of small insects such as ants, spiders, and beetles as well as occasional small lizards, frogs, or rodents [2,3]. This varied diet provides a vast nutritional diversity and intake that may offer natural oils or similar substances that work in a similar method as gastrointestinal lubricants [14,31]. These lubricants (natural or synthesised) propel ingested strands of hair from the stomach into the duodenum [14]. This prevents accumulation of material in the pylorus, which may lead to a blockage of the GIT. Paraffin oil is a commonly used domestic feline gastrointestinal lubricant as it is tasteless, odourless, and can reduce the occurrence of obstruction and trichobezoar formation [14]. It has been a successful dietary addition for other species [22] in preventing these issues. In this case series study, it appears to likely also play a similar role in preventing trichobezoar occurrence in dunnarts with study protocols, aetiology, and treatments extrapolated from other species.

The nutritional composition of the diet must be considered when assessing the risk of trichobezoars [28]. However, it remains unverified if the nutritional profile of current diets fed in captivity are the most appropriate diet for dunnarts. It is well documented that diets lacking fibre or containing excess protein can predispose cats [14,27,28] and rabbits [19,20,30] to trichobezoars. By correcting the ratios of these components in the diet, several studies have described a decrease in trichobezoars [28,29]. Increased fibre typically improves peristalsis throughout the GIT and can ultimately decrease the hair entanglements that can lead to trichobezoar formation [28]. It has also been proposed that higher fibre intake may aid in the elimination of single strands of hair from the GIT, further inhibiting trichobezoar accumulations [28,29]. Further research is necessary to identify the ideal diet of fat-tailed dunnarts, to adequately meet nutritional requirements and physiological GIT functions of this species.

### 4.2. Behaviour and Environmental Considerations

It is important to consider the elements of captivity that may be contributing to trichobezoar occurrence. Dunnarts are a solitary species and therefore the design of their enclosure plays a fundamental role in allowing natural behaviours to be maintained [3,32]. For example, the ability to exercise at night, given that they are a nocturnal species, can be a useful technique to support dunnart natural nocturnal behaviours [3,32]. Additionally, their natural habitat covers an extensive home range and, thus, the size and shape of their enclosures (and enrichment offered) must provide sufficient space to carry out routine activities [4]. According to the Australian Department of Education Animal Ethics Committee standard operating procedures (SOPs) (2020), each dunnart should have a minimum cage area of 0.25 m^2^. Enclosures should be fitted with hollow logs for shelter, with enrichment offered (e.g., toilet rolls and/or empty egg cartons) and choice of shelter. While the facility offered these items, it appeared that these aforementioned items alone were insufficient to reduce the presence of trichobezoars. The additional measures of paraffin and logs to promote shedding were implemented and have likely played a role in successfully preventing further trichobezoar formation. It has been noted that in other species, reduced or limited access to enrichment or space to promote natural behaviours and exercise may lead to decreased gastrointestinal motility and thereby potentially increase the risk of gastrointestinal obstruction [17,19]. It appears that in addition to the recommendations by the Australian Department of Education SOPs (2020), that further environmental enrichment is likely necessary to promote a more natural environment that could help avoid trichobezoar formation.

Following winter, and as the weather begins to warm up, many species undergo a natural moult [20]. During this time period (spring), animals can be exposed to higher risk of excess fur ingestion [12,20,28]. In cats, it has been reported this risk can be mitigated through daily brushing and a higher incidence in cats with long hair [14]. The natural environment of dunnarts indicates that moulting fur is likely displaced from the body via airflow and contact with vegetation amidst the shrublands and grasslands they inhabit [1]. While this colony is kept underground and on a continuous 12:12: (light:dark) cycle, it might be possible that seasonal effects may still affect the colony. The extra addition of autoclaved bark to the captive enclosures appeared to potentially assist in creating an environment more representative of the wild environment to facilitate the removal of moulting hair. In addition, related environmental enrichment and paraffin oil appeared to have likely played a role in preventing further deaths in the colony (since October 2021).

Overgrooming can be a common occurrence for animals in domesticated and captive populations [12,14]. It may indicate an underlying medical, parasitic, and/or behavioural condition [12,13,14,20]. Excess chewing or licking of affected areas can lead to hair loss, excess ingestion, and trichobezoar formation [14,16,33]. There was no evidence indicating that pruritic or pathological parasitic infections contributed to the development of trichobezoars in this population. However, animals’ co-inhabiting enclosures may also be more susceptible to excess fur ingestion through co-grooming of conspecifics [12]. Co-grooming often occurs in dams with young and must be well managed to minimise hair ingestion [20], which did not appear to be a factor with these affected dunnarts. Spot cleaning of fur from nesting boxes and enclosure furnishings may be beneficial during these time periods to reduce excess fur in the environment and to promote shedding of fur onto enclosure furnishings.

Animals experiencing negative states from stress (distress), boredom, or anxiety may overgroom as a maladaptive behaviour, leading to excess fur ingestion [12,16,29,33]. Stress can also lead to a decrease in gastrointestinal motility [14,22]. Therefore, it is integral that stress in captivity is reduced by keeping handling to a minimum and providing environments that allow animals to express natural behaviours [16]. Some work has been undertaken to improve the husbandry and management of dunnarts [7,8] which likely played an important role in eliminating further deaths due to trichobezoars (all deaths are now necropsied). However, further work into dunnart enclosures, diet, and other husbandry-related practices would be welcomed.

### 4.3. Alternative Treatments

The primary factor preventing the development of trichobezoars in other species is ensuring normal gastrointestinal function. This includes providing adequate hydration, minimising stress, and allowing animals adequate space to exercise [17,19,30]. Other proposed treatment options are diet modifications, including additional enzymes as well as changes in feeding regimes that may increase gastrointestinal motility [14,26,28,29,30]. It is not known whether enzymes may be beneficial to the gastrointestinal system of dunnarts, and this is something that could be considered for future exploration.

The vast majority of trichobezoar preventative research discusses the inclusion of various substrates in diets to prevent trichobezoar formation. Howell et al. [26] considered the use of cellulose in the diets of the cotton rat as a preventative measure. The addition of insoluble fibre in diets is aimed at increasing the rate of passage of particles through the GIT. Whilst this strategy does decrease the occurrence of trichobezoars, it does not fully prevent their formation [26,28]. Subsequently, Miltenburg et al. [29] trialled sugarcane as a possible fibre source. However, the results of this study were inconsistent, with individuals showing variable results to treatment. A later study by Loureiro et al. [28] evaluated the benefits of beet pulp as a fibre source in feline diets fed to domestic cats. It was shown that the added fibre increased the gastrointestinal passage time and subsequent faecal excretion but did not reduce the size or number of trichobezoars found in the study animals. The small but consistent body of literature analysing fibre content in various species suggests this may be an important consideration when formulating the ideal dunnart diet.

It has been proposed that the addition of pineapple juice or an alternative proteolytic enzyme to rabbit diets may be beneficial in breaking down the protein matrix that holds the trichobezoars together [30]. However, this theory does not yet have substantial evidence and should be used with caution. Another study suggests that meal size can play an important role in gastric motility [14]. Animals fed smaller, more frequent meals were found to have quicker gastric emptying [14]. Dunnarts in this study were fed once daily their entire ration to enable grazing of adequate intake throughout the day; therefore, it is unknown if more frequent feeding might prove beneficial. However, this species can consume their own body weight in food each night and is thought to have a rapid digestion time (<1 h) with frequent defaecation observed [8].

### 4.4. Study Limitations

Of the 22 recorded deaths over 4 years from an estimated ≥ 250 dunnarts, 13 animals were not necropsied with no obvious cause of death determined. This is a significant limitation of the study, and therefore the true number of trichobezoar-related issues may be underreported. Consequently, retrospective epidemiological incidence and frequency of this issue cannot be utilised. Additionally, trichobezoar diagnosis was only undertaken after death and any incidence of subclinical trichobezoars in the colony was unknown. Overall, given the small number (*n* = 3) of trichobezoars observed, it was not possible to analyse trends in signalment, incidence, and possible seasonality of cases.

The modifications to the management protocols discussed in this study were first implemented from September and October 2021. As discussed earlier, this is the season when trichobezoar pathology is most likely to occur (Australian spring). Although this population was housed indoors with constant yearly light cycles, it is thought that they might still be innately aware of the natural seasons and continued to undergo some seasonal behaviours. As of April 2025, it appears that the preventative management protocols have been successful as no further instances of trichobezoars have been recorded in this colony with over 2000 animals produced or entering the colony over this time.

Continued research into trichobezoars, husbandry, and management could be useful to provide additional beneficial refinements for captive dunnarts. Further areas for exploration could include evaluating which genetic, environmental, or other factors could predispose animals to excessive hair ingestion or similar factors that promote the formation of trichobezoars. Similar to cats and other species, studies could be undertaken to investigate alternative diets for captive dunnarts to determine if the inclusion of more fibre and/or other nutrients may offer additional health and/or anti-trichobezoar formation benefits.

## 5. Conclusions

Like many other species, dunnarts may be more susceptible to trichobezoars when housed in captivity. The addition of an intestinal lubricant alongside purposeful enrichment can be options to consider for minimising the risk of occurrence. It is also important to ensure that potential stressors are assessed and minimised to maintain adequate gastrointestinal function. It is integral that any sudden death of small marsupials is formally necropsied to determine the cause of death and that trichobezoars are considered as a differential diagnosis (now performed in the colony). The interventions investigated in this study appeared to be successful with over 2000 animals since entering or being born into the colony without any trichobezoars. We suggest that it would be useful for other research and conservation institutions to review if incorporating these techniques could also potentially improve the welfare of other dunnarts and small marsupials when housed in captive environments to avoid trichobezoar formation.

## Figures and Tables

**Table 1:** Dunnarts found dead with pathology resembling trichobezoars.

Date of Death	Animal Number	Sex	Age	Pathology
7 November 2020	My291	Male	1 year 4 months	Furball lodged in small intestine
27 August 2021	868	Female	0 years 8 months	Furball
4 October 2021	768	Female	1 year 7 months	Furball in stomach

## Data Availability

Data can be accessed by contacting the authors.

## Appendix A

### Diet

The dunnart diet was made in large batches able to feed 100 animals for 2–3 days.

Ingredients:
  - 500 g Wombaroo (Glen Osmond, South Australia, Australia) small carnivore diet.
  - 400 g of Advance Kitten (Bathurst, New South Wales, Australia) dry cat biscuits—chicken
  - 2× tins (156 g each) of Hills AD (Sydney, New South Wales, Australia) adult cat science diet

(if Hills AD is unavailable, replace with two cans of Royal Canin (Melbourne, Victoria, Australia)—recovery feline/canine diet)

- 100 mL Wombaroo (Glen Osmond, South Australia, Australia) —The Good Oil
- 15 mL Vetsense Paraffin Oil (Mulgrave, Victoria, Australia)
- 2 L of boiling hot water, let dry biscuits soak for at least an hour as this will allow the biscuit to swell (add more water if necessary).

Instructions:

Thoroughly hand mix wearing surgical gloves for sterility.

Each animal is given, 5 g/per day (approximately 1 teaspoon each), this portion is doubled on the Saturday feed.

When the animals are checked on the Sunday, bowls were topped up if low.

The wet mix is increased to 30 g/per animal/per day when females have young.

Provide two portions of 5× meal worms and one portion of 3× crickets per animal weekly.

Mums with pouch young require 10 mealworms per feed 3 times a week and the wet diet increased while the young grow and start to wean off their mum.

2 drops of Pentavite were added to each water sipper/tube.

## Appendix B

### Raw Data

**Table A1:** Raw data of all deaths recorded over the duration of the study.

Date	Animal ID	Sex	Necropsied	Pathology
16 April 2018	Mg57	Male	NO	
14 May 2018	FG118	Female	YES	Organs looked liquified
24 May 2018	My100	Male	NO	
18 December 2018	Fr155	Female	NO	
11 March 2019	Mg189	Male	YES	No abnormalities detected
8 April 2019	Mr239	Male	NO	
14 April 2019	Mg189	Male	YES	No abnormalities detected
3 June 2019	Mr240	Male	NO	
6 August 2019	Fw262	Female	NO	
21 August 2019	Fb199	Female	NO	
8 September 2019	Fy249	Female	YES	Enlarged uterus
9 December 2019	Mg244	Male	NO	
10 April 2020	Fr258	Female	NO	
10 September 2020	My300	Male	NO	
7 November 2020	My291	Male	YES	2 cm furball lodged in small intestine
28 November 2020	Mg311	Male	NO	
16 December 2020	F760	Female	NO	
6 January 2021		Female	NO	
27 May 2021		Female	NO	
27 August 2021		Female	YES	Gastrointestinal tract blockage unknown size
4 October 2021	768	Female	YES	Trichobezoar in stomach unknown size
8 December 2021		Male	NO	
